# Changes of Plantar Pressure and Gait Parameters in Children with Mild Cerebral Palsy Who Used a Customized External Strap Orthosis: A Crossover Study

**DOI:** 10.1155/2015/813942

**Published:** 2015-11-10

**Authors:** Wen-Dien Chang, Nai-Jen Chang, Hung-Yu Lin, Ping-Tung Lai

**Affiliations:** ^1^Department of Sports Medicine, China Medical University, No. 91, Hsueh-Shih Road, Taichung City 404402, Taiwan; ^2^Department of Sports Medicine, Kaohsiung Medical University, No. 100, Shih-Chuan 1st Road, Kaohsiung City 80708, Taiwan; ^3^Department of Occupational Therapy, I-Shou University, No. 8, Yida Road, Jiaosu Village, Yanchao District, Kaohsiung City 82445, Taiwan; ^4^Department of Physical Therapy and Rehabilitation, Rehabilitation Assistive Device Center, Da-Chien General Hospital, No. 6, Shin Guang Street, Miaoli City 36049, Taiwan

## Abstract

Toe-in gait and crouch gait can make children with mild cerebral palsy fall and suffer improper balance during walking or ambulation training. A customized external strap orthosis for correcting leg alignment was used to resolve this problem. The purpose of this study was to research the immediate effects while wearing the customized external strap orthosis. Pressure platform was used to assess the plantar pressure through static and dynamic assessments and to record the changes in path of pressure trajectory. Motion image analysis system was used to record the gait parameters, which included gait speed, stride length, and cadence. The influence of both wearing and removing the orthosis on the dominant leg of children with mild cerebral palsy was analyzed. Nine children with mild cerebral palsy, who all had a dominant right leg, were recruited. After wearing the orthosis, all gait parameters improved, and foot motion changed in the stance phase of the gait cycle. The path of pressure trajectory closing to the midline was also observed during dynamic assessment. Changes in plantar pressure and path of pressure trajectory were observed and the orthosis device could provide immediate assistance to correct the leg alignment and improve the gait performance in children with mild cerebral palsy.

## 1. Introduction

The cause of cerebral palsy in children occurs from damage to the brain in early childhood [1]. It is associated with several symptoms that affect the nervous and skeletal systems, such as spasticity, contracture, and uncoordinated movement, and that can affect movement ability [1, 2]. Cerebral palsy also affects the senses, cognition, and language and has a severe impact on children [3]. The patients recover slowly from it because it is nonprogressive damage to the brain [2]. The prevalence of cerebral palsy ranges from 1.5 to 2.5 per 1000 live births among Western countries [4], and many of the children with this neuromuscular disease experience dysfunction in their growth. Muscular spasticity in the lower limbs can lead to toe-in gait and crouch gait, resulting in falls and the inability to balance during walking and ambulation training [5].

Hip and knee joint provide dynamic and static stabilities during the gait cycle. Medial femoral torsion, which is caused by abnormal muscle tension, affects toe-in gait and crouch gait and increases instability during ambulation [6]. Toe-in gait and crouch gait decrease the base of support in the stance phase and increase legs crossed collision in the swinging phase of gait, which may increase the risk of falling [7]. The maldirected lower extremity reduces muscle action and decreases gait efficiency [8]. Physical therapists conducted children with cerebral palsy from the sagittal plane to correct toe-in gait and crouch gait. They often used a solid ground reaction ankle foot orthosis, which is formed from polypropylene and was a molded plastic and custom fabricated orthosis. It could increase ankle dorsiflexion and hip and knee flexion during the stance phase of gait and was used more commonly in children with cerebral palsy [9]. Currently, transverse plane corrective orthoses are provided, such as TheraTogs, which is one kind of dynamic elastomeric fabric orthoses and could increase lateral torque in gait [10]. This orthosis for children with cerebral palsy or users was useful, but it required long-term use and complicated adjustment. When the necessity of lower limb motion correction and fall prevention occurred in a short-term treatment course, physical therapist needs facilitation and assistance of a specific orthosis, which can provide lateral torque to apply for immediate use. Therefore, the customized external strap orthosis was designed for children with cerebral palsy to correct leg alignment.

Foot plantar pressure was often used to observe abnormal lower limb alignment and to determine body weight across the lower limbs [11, 12]. When the lower limbs exhibit abnormalities in movement, numerous studies have reported the changes in plantar pressure, surface contact area, and body weight distribution under dynamic and static conditions [11]. Plantar pressure reliably indicated problems of lower limb alignment and can be used as a tool to assess toe-in gait in a child with cerebral palsy [13]. Some studies indicated that outcomes of using lower extremities orthoses could present the changes of spatiotemporal gait analysis and represent the improvement of gait function [9, 10]. The current report is a preliminary study exploring the changes in plantar pressure and spatiotemporal gait parameters (gait speed, cadence, and stride length) while using the proposed customized external strap orthosis. The immediate changes in kinetics of the customized external strap orthosis were still unclear. Therefore, we focused on the differences in plantar pressure and gait parameters and compared the changes in foot function and path of pressure trajectory of plantar pressure, while children with mild cerebral palsy wore the customized external strap orthosis.

## 2. Materials and Methods

### 2.1. Study Design and Participants

The current study was a randomized crossover design and was conducted to examine the changes in the plantar pressure of children with mild cerebral palsy, who wore a customized external strap orthosis compared with removing the orthosis. The study procedure was approved by the medical ethics committee of a hospital, and the parents of children gave informed consent for study process after being informed of the study. Participants, who were children with mild cerebral palsy, were recruited in a child development rehabilitation center of a hospital in Taiwan between August 2012 and July 2014. The inclusion criteria were confirmed assessments of toe-in gait gross, physical examinations, such as a foot progression angle, external tibial torsion angle, and femoral anteversion angle, and motor function classification system (GMFCS) scores I and II that could understand the test's instructions. All of them were assessed and recorded by a physician and physical therapist. Exclusion criteria were that they had lower extremity surgeries, such as osteotomy or subtalar joint lengthening surgery, language or hearing impairment, and difficult standing and walking without wearing shoe or orthoses. As this is a preliminary study, the customized external strap orthosis was first applied for children with cerebral palsy. Thus, estimated sample size used the outcomes based on previous study of Jagadamma et al. [14] on children with cerebral palsy, who wore an ankle foot orthosis. They detected a medium effect size on kinetic changes during stance at a *p* = 0.05 and power = 80%, and estimated sample size was 5 children per group. So, in the current study, the sample size was set at 9 children.

### 2.2. Customized External Strap Orthosis

**Figure 1:**
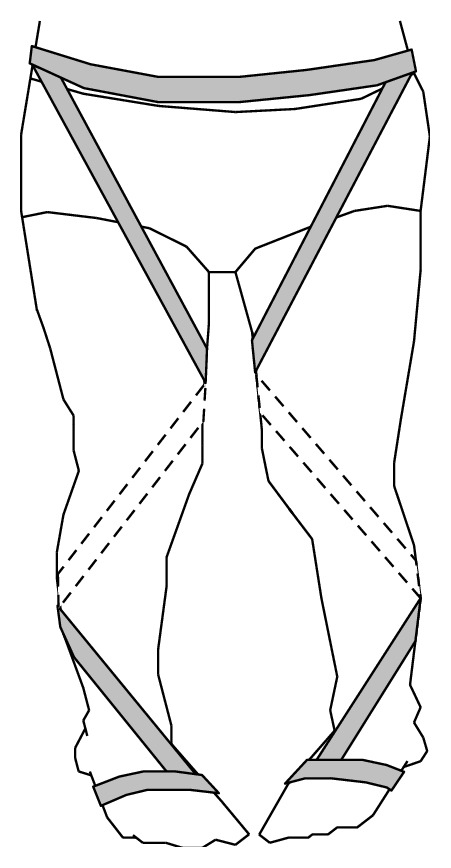
Customized external strap orthosis.

The customized external strap orthoses (Patent number M465892, Taiwan) were manufactured in a rehabilitation center in a hospital and adjusted for each child by an occupational therapist. The orthoses were fabricated from an elastic fabric strap and a metal retaining clip with a loop fixed to the ankle and foot (Figure 1). The strap was tensioned with a spiral pattern, which ran from the lateral ankle to posterior calf and inner thigh to lateral sacral crest, and was fixed to be close to anterior superior iliac spines. It was not wrapped around the hip and knee joints. The customized external strap orthosis formed an outward strap and provided an elastic force that can assist in correcting the child's lower extremity. In this study, the children wore the orthosis on both lower extremities and removed the shoe and other orthoses during the test.

### 2.3. Outcome Measures

A piezoresistive pressure platform (EPS/C1, Loran Engineering, Italy) was used to record the changes in plantar pressure during walking. The pressure plate was comprised of 4,096 points of pressure sensors, and the sampling frequency was set at 100 Hz. We analyzed the changes in plantar pressure while wearing or removing the customized external strap orthosis. Both feet were tested on the pressure platform, and we adopted the data of the dominant leg for analysis. The interval between the two assessments was 30 min and three repetitions of the test were completed. The static assessment required subjects to relax in a standing position, to look straight ahead, and to stand on the pressure plate for 1 min [11, 12, 15]. The data from plantar pressure distribution while standing were collected. The children were then asked to walk forward on the pressure plate and to go back and forth in three tests for dynamic assessment, from which we chose the optimal 2-step method for analysis [16]. The children were tested wearing or removing the customized external strap orthosis in both assessments.

As receiving original data of plantar pressure, Biomech 4.0 software (Loran Engineering, Italy) was used for analysis. The area of plantar pressure was divided into 10 regions including the first toe (T1), second to fifth toes (T2–5), first metatarsal (M1), second metatarsal (M2), third metatarsal (M3), fourth metatarsal (M4), fifth metatarsal (M5), midfoot (MF), medial heel (MH), and lateral heel (LH) [11]. The peak force and loading area of foot were recorded as well as assessing mean peak planter pressure of 10 areas, which represented the plantar loading of different area of foot. During dynamic assessment, the indexes of heel rotation and foot balance were calculated from the mean peak planter pressure of specific regions, which were recorded continuously in the tested phase. The formulas used were heel rotation index = MH – LH and foot balance index = (M1 + M2 + HM) − (M3 + M4 + M5 + HL) [17]. Positive values of heel rotation index represented movement toward heel valgus and negative values were toward heel varus. The value of foot balance index represented the stability of the total foot. Positive values of foot balance index showed movement toward foot pronation but negative values of foot balance index showed movement toward foot supination. The center of pressure trajectory, heel rotation index, and foot balance index were evaluated from dynamic assessment of motion trajectory. The path of pressure trajectory was analyzed, and anteroposterior index of pressure trajectory (anteroposterior length of the path of the pressure trajectory divided by anteroposterior length of the foot) and excursion index of pressure trajectory (maximum lateral deviation of pressure trajectory divided by maximum width of the foot) were calculated [13, 18]. Reliable plantar pressure data, which had intraclass correlation coefficients of 0.5–0.9 in all regions of the foot, can be collected in children [19].

Gait analysis required the children with cerebral palsy to walk on a treadmill and treadmill speed was set at a comfortable rate for them. A physical therapist and caregivers stood at left side of children to help him or her avoid injury during the test. Three 10 min tests and 5 min interval breaks were completed, in order to avoid the phenomenon of fatigue occurrence. The motion image analysis system (TEMPLO motion analysis, Contemplas, Germany) was used to analyze the spatiotemporal influence of gait, and a 150 fps camera was set up to the right side of the treadmill to capture synchronous images. The analysis system had high test-retest reliability (ICC 0.72–0.91) and was valid for measuring the motion of lower extremity [20]. Reflective markers (9 mm diameter) were fixed on bony landmarks of the right lower extremity (greater trochanter and lateral femoral condyle of the femur and lateral malleolus of the tibia). Using the reflective markers to automatically catch reflected points on the successive images, the gait speed, cadence, and stride length of gait parameters were calculated and analyzed. The assessments of plantar pressure and gait analysis were recorded by a physical therapist, and the extractive data were blinded to be analyzed by another examiner.

### 2.4. Statistical Analysis

Data was analyzed using descriptive statistics in SPSS 15.0 (SPSS Inc., Chicago, IL, USA) to compare the continuous variables. A paired *t*-test was used to compare the mean peak planter pressure in the 10 regions of foot, the anteroposterior index and excursion index of the pressure trajectory, and the gait parameters with and without wearing the customized external strap orthosis. A two-tailed *p* value of less than 0.05 was statistically significant.

## 3. Results

**Figure 2:**
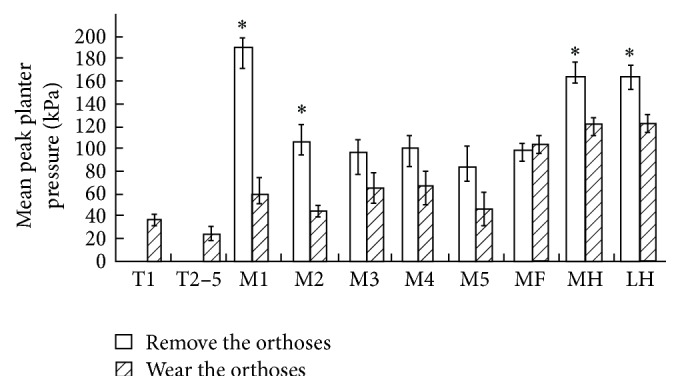
The mean peak planter pressure of 10 areas of foot (T1: first toe; T2–5: second to fifth toes; M1: first metatarsal; M2: second metatarsal; M3: third metatarsal; M4: fourth metatarsal; M5: fifth metatarsal; MF: midfoot; MH: medial heel; LH: lateral heel); ^*∗*^ *p* < 0.05.

Nine children with mild cerebral palsy (age = 8.73 ± 2.14 years; weight = 25.61 ± 1.38 kg; height = 120.82 ± 2.77 cm), who all had a dominant right leg, were recruited and performed the short distance stride repetitions while wearing and removing the customized external strap orthosis. All of them received physical therapy in the rehabilitation center. They had experiences of wearing ankle foot orthosis and wore customized external strap orthosis for the first time. According to Staheli et al., no significant flexion contracture and rotational malalignment of the knee and hip were found [21], because external tibial torsion angle was 25°–35° and the femoral anteversion angle was 20°–30° in all children. In Table 1, the results show that there were statistically significant differences between wearing and removing the customized external strap orthosis in all gait parameters (*p* < 0.05). In the static assessment, the mean peak planter pressure on the pressure platform for the children while removing the orthosis was centered on M1, M2, MH, and LH. After wearing the customized external strap orthosis, the loading pressure shifted to the areas of T1 and T2–5 (Figure 2). A significant difference in mean peak planter pressure of M1, MH, and LH was observed between wearing and removing the orthosis (*p* < 0.05).

**Figure 3:**
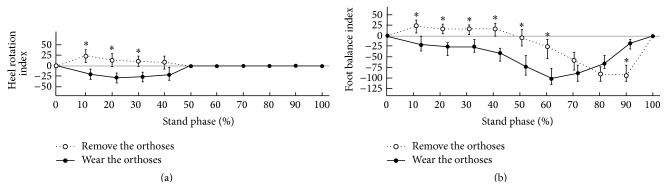
The heel rotation (a) and foot balance indexes (b) under wearing and removing the customized external strap orthosis; ^*∗*^ *p* < 0.05.

**Figure 4:**
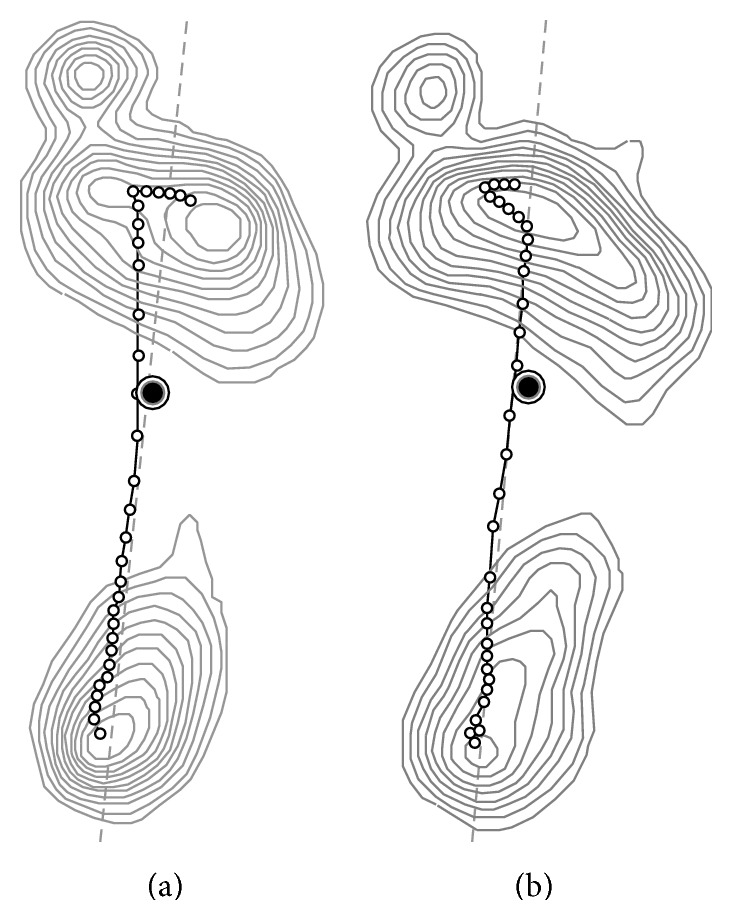
The path of pressure trajectory, which is represented as a curved line, under removing (a) and wearing (b) the customized external strap orthosis. A straight line is represented as a horizontal line in walking distance, and closed curves represented plantar pressure gradient.

In the values of heel rotation index of dynamic assessment, the graphic wave was represented differently in 0% to 50% of the standing phase (Figure 3(a)). The values of heel rotation index were positive while removing the customized external strap orthosis, whereas its values were negative while wearing the orthosis. The values of heel rotation index were 0% above 50% of the standing phase because the heel was off. Its values at 10% to 30% when removing the customized external strap orthosis were higher than those when the orthosis was worn, and significant differences were observed between wearing and removing the orthosis (*p* < 0.05).  Figure 3(b) shows that for 0% to 70% of the standing phase the values of foot balance index when wearing the customized external strap orthosis were lower than those without wearing the orthosis. However, the results were opposite for 80% to 100% of the standing phase. Significant differences were observed in foot balance index for 10% to 60% and 90% of the standing phase (*p* < 0.05). The graphic trough moved forward from 90% to 60% of the standing phase after wearing the customized external strap orthosis. Comparing with wearing and removing the orthosis, the anteroposterior index and excursion index of pressure trajectory increased (Table 1). The path of pressure trajectory was close to the foot midline, and the peak plantar pressure response changed from the heel to the forefoot (Figure 4).

## 4. Discussion

Cerebral palsy causes nervous system injury and results in lower limb spasticity, contracture, lack of coordination, muscle weakness, and inability to move independently in children [2, 22]. When comparing the number of motor units recruited during a voluntary contraction, the children with cerebral palsy had less units than the healthy children [1]. Along with muscular spasm and joint contracture, the problems cause abnormal gait, joint restriction, and loss of functional activity [23]. Ambulation is an important functional activity, as a previous study of Kadhim and Miller [23] pointed out that 90% of children with cerebral palsy had instability problems during gait, and 54% of the children were unable to walk alone. Therefore, the children with cerebral palsy needed more training and assistance for walking in the rehabilitation treatment. Uden and Kumar [24] indicated that metatarsal valgus, excessive femoral anteversion, tibial medial torsion, and muscular weakness of the lower extremity resulted in the case of toe-in affected gait pattern. The ankle foot orthoses are widely used for children with cerebral palsy and could improve the gait efficiency [25]. The orthosis structure, which restricts range of motion of ankle, could increase the ankle joint stability and position control [9]. In spatiotemporal findings of the study of Buckon et al. [26], a solid ground reaction ankle foot orthosis for children with cerebral palsy produced a significant increase of 0.06 m in stride length, but decreases of 0.04 m/s and 18 steps/min in gait speed and cadence, respectively. The results of Hayek et al. [27] showed that while wearing the ankle foot orthosis, stride length and gait speed of children with diplegic cerebral palsy were significantly increased by 0.12 m and 0.11 m/min, respectively. Abd El-Kafy [28] found that there were no statistically significant differences in gait speed, cadence, and stride length compared with wearing and removing the ankle foot orthosis. The systematic review results of Ridgewell et al. [29] represented that increases in stride length were common, and the gait speed and cadence were less predictable for children with cerebral palsy wearing ankle foot. However, our results showed that customized external strap orthosis can improve gait abilities, in addition to significantly improving gait speed (increase of 1.67 m/min), cadence (increase of 2.48 steps/min), and stride length (increase of 0.02 m). The reason may be that the applied external rotation force can correct the maldirected lower extremity and increase stability in the gait cycle. This result was the same as the outcomes of previous studies [10, 14], which applied dynamic elastomeric fabric orthoses on children with cerebral palsy. These orthoses, which are close-fitting garments, cost almost the same as solid ground reaction ankle foot orthoses, and the children need to take a long time to wear them. Previous study found that the orthoses could correct static posture control but cannot improve dynamic balance control in a 6-week timeframe [30]. However, inexpensive and convenient customized external strap orthosis can achieve immediate corrective effects of the static and dynamic aspects in the children with mild cerebral palsy.

Treadmills are devices often used not only as a gait evaluation tool but also as rehabilitation training tools for patients with stroke, spinal cord injuries, and cerebral palsy [31]. Previous study showed that children with cerebral palsy can improve their walking distance, speed, and standing posture by treadmill training [22]. Also, clinical providers often use treadmill to train lower extremity gait in children with cerebral palsy. There are both advantages and disadvantages to using the treadmill for training. Advantages are that it allows execution and observation of the children's gait cycle in a limited space. In addition, the walking speed and distance can be controlled freely in accordance with the children or environment requirements. Moreover, the treadmill makes it possible for children to continue ongoing activities during the time of the operation, which is convenient for researchers to collect gait parameters [31]. The disadvantage is that the muscles of lower extremity may be passively driven by treadmill motor. The children cannot interact with the environment, so that they decrease the experience to improve their motor control, which is a process to determine suitable muscle forces and joint activation to walk on the ground. In addition, the treadmill is relatively flat-surfaced and stable and is different from uneven ground [32]. Therefore, it is easier for the children to control their balance on treadmills [31, 32]. Gait strategy on a treadmill is different from that on the ground, and, consequently, generalization on the ambulation skill learning transfer from walking on a treadmill to walking on the ground is difficult for the disabled. But for children with cerebral palsy, the treadmill is mainly used to practice their ability to walk and to improve their ability to walk in their daily life [32]. So, observation of walking performance on the treadmill can help therapists to assess the outcome of ambulation training with a new use of orthoses, as well as indirectly observe their ability to walk on the ground. Our findings also suggest that the customized external strap orthosis could improve the gait performance on the treadmill in children with cerebral palsy.

Edelstein [33] believed that ankle foot orthoses, crutches, and other aids can help children with cerebral palsy to improve balance, assist acceleration and deceleration of displacement, and reduce the weight load on the body. Therefore, the use of walking aids is very important for children with cerebral palsy when walking. In previous study, solid ground reaction ankle foot orthoses have been used to help children with cerebral palsy to walk [9]. Solid ground reaction ankle foot orthoses are devices designed to increase the knee extension. They also limited ankle dorsiflexion and therefore increase the stability of the lower limbs by changing the ground reaction forces [34]. Lucareli et al. [22] found that solid ground reaction ankle foot orthoses can change the knee and ankle joint dorsiflexion in gait sagittal plane during stance phase in children with spastic cerebral palsy. However, the design concept of solid ground reaction ankle foot orthoses is to improve stance phase of gait by restricting ankle dorsiflexion. This principle is different from the customized external strapping orthosis, which lacks cross section support in external rotation strength. With regard to customized external strapping orthoses, the orthosis assists the gait of children with cerebral palsy by utilization of elastic-induced external rotation movements of lower extremity. The results of the present study showed that wearing customized external strapping orthoses could enhance the ability to walk on a treadmill, which is reflected by the significant differences in gait speed, cadence, and stride length. Thus, customized external strapping orthoses are not only less restrictive to the joints while walking for children with cerebral palsy, but also more convenient for the therapist to use them in walking training on the treadmill.

Enhancing the stability of the lower extremity during the stance phase of the gait cycle is critical. This allows the contralateral foot to perform a smooth and swinging motion [35]. A stable closed kinetic chain can increase stability during walking and decrease the risk of falling [35]. Past study had revealed that patients with neuromuscular diseases, such as cerebral palsy, stroke, and Parkinson's disease, have a high risk of falling [36]. Regardless of whether it is from an activity of daily living or rehabilitation training, fall prevention is critical [37]. Numerous assistive devices for the lower extremities assist the patients in increasing stability when walking [38]; however, excessive stability may restrict normal movement, and this is particularly crucial for active children [39]. The customized external strap orthosis can change the alignment of the lower extremity and decrease loading on plantar pressure. Because the orthosis has an elastic property, it provides a corrective force over the lower extremities. Compared with other orthoses, such as ankle foot orthosis and ankle splints, the customized external strap orthosis is more suitable for children with mild cerebral palsy to use.

To the best of our knowledge, it is difficult to use a pressure platform to analyze foot pressure while wearing ankle foot orthoses in a kinetic analysis. This is the first use of static and dynamic assessments to compare the change in plantar pressure, while children with mild cerebral palsy used a customized external strap orthosis. The results of the current study showed that plantar pressure was distributed among 10 areas and increased in the T1 and T2–5 regions while undergoing static assessment. Plantar pressures were distributed to the forefoot area after wearing the customized external strap orthosis. The elasticity of the customized external strap orthosis pulled the foot so that it rotated externally and the biomechanical mechanism maintained the postural balance. This result can facilitate medial rotator muscles of lower extremity and increase the area of toe contact. Increased contact with forefoot regions can improve standing stability and increase the base of support [40]. The findings of static assessment also represented that plantar pressure distribution decreased practically in all the analyzed regions except in the T1 and T2–5 regions, while the children wore the orthoses. Recording plantar pressure data from the dominant foot is a common clinical evaluative method [19]. When the children removed the orthoses to test the pressure platform, physical therapist or caregivers standing on nondominant side of the children paid close attention to them. Increase of environmental interference while removing the orthoses and improvement of posture control while wearing the orthoses were the reasons of decrease of plantar loading in the dominant foot. In the dynamic assessment, the results indicated that foot supination occurred while the dominant foot moved from heel contact to midstance in the gait cycle. Foot balance index represented the time that foot supination was advanced from heel contact of the stride [18]. The current study demonstrated that the lower extremity was corrected by rotating externally and the foot remained in supination while the children wore the customized external strap orthosis. The phenomenon was observed in foot midstance and continued in the stance phase. The path of pressure trajectory also showed that weight shift was close to the midline and moved to the lateral side, and the change was meant to increase postural stability during weight loading transition [41]. This result had occurred because of the elasticity of the customized external strap orthosis. The use of the orthosis was supported to improve lateral leg rotation during strides and inferred to reduce the risk of falling.

The present study is the first to use the customized external strap orthosis for children with mild cerebral palsy that assists in standing and walking. In addition, the changes in plantar pressure and increases in gait parameters and excursion index of pressure trajectory were observed. The cost of customized external strap orthosis was lower than that of ankle foot orthosis or dynamic elastomeric fabric orthoses. In clinical practice, the inexpensive customized external strap orthosis had potential utility for the children with mild cerebral palsy. We concluded that the orthosis positively affects kinetic changes in the lower extremities. However, there were some limitations of the present study. First, it lacked participants with other diagnosed kinds of cerebral palsy, and the sample size was still small. Therefore, the data did not accurately represent the outcome for typical pathological gait in children with cerebral palsy. Second, the length of customized external strap orthosis must be defined by the individual leg length in the children. The variation of elasticity should be considered further in a future study. Third, long-term effects of the orthosis on functional activity also needed to be investigated.

## 5. Conclusion

In the current study, the findings of plantar pressure and spatiotemporal gait analysis indicated the potential use of customized external strap orthosis to improve the gait speed, stride length, and cadence and influence the foot motion and path of pressure trajectory in the children with mild cerebral palsy. The elastic force of the orthosis can provide immediate assistance to correct the leg alignment and improve the gait performance. The current results support the importance of applying a customized external strap orthosis for the child in the treadmill ambulation training.

## Figures and Tables

**Table 1:** Changes in the gait parameters and path of pressure trajectory.

	Removing the orthoses (*n* = 9)	Wearing the orthoses (*n* = 9)	*F*-value	*p* value
Gait speed (m/min)	41.66 ± 1.73	43.33 ± 1.32	0.25	0.036^*∗*^
Stride length (m)	0.62 ± 0.02	0.65 ± 0.02	0.45	0.011^*∗*^
Cadence (steps/min)	60.22 ± 1.93	62.67 ± 1.22	2.22	0.005^*∗*^
Pressure trajectory				
Anteroposterior index (%)	59.67 ± 6.59	64.55 ± 3.78	3.17	0.076
Excursion index (%)	15.56 ± 1.94	19.23 ± 3.32	1.14	0.013^*∗*^

^*∗*^ *p* < 0.05, removing the orthoses versus wearing the orthoses.
